# Recent advances and emerging therapies in anaplastic thyroid carcinoma

**DOI:** 10.12688/f1000research.13124.1

**Published:** 2018-01-18

**Authors:** Maria E. Cabanillas, Mark Zafereo, Michelle D. Williams, Renata Ferrarotto, Ramona Dadu, Neil Gross, G. Brandon Gunn, Heath Skinner, Gilbert Cote, Horiana B. Grosu, Priyanka Iyer, Naifa L. Busaidy

**Affiliations:** 1 Department of Endocrine Neoplasia & Hormonal Disorders, The University of Texas MD Anderson Cancer Center, 1515 Holcombe Blvd, Houston, TX, 77030, USA; 2 Department of Head and Neck Surgery, The University of Texas MD Anderson Cancer Center, 1515 Holcombe Blvd, Houston, TX, 77030, USA; 3 Department of Pathology, The University of Texas MD Anderson Cancer Center, 1515 Holcombe Blvd, Houston, TX, 77030, USA; 4 Department of Thoracic Head and Neck Medical Oncology, The University of Texas MD Anderson Cancer Center, 515 Holcombe Blvd, Houston, TX, 77030, USA; 5 Department of Radiation Oncology, The University of Texas MD Anderson Cancer Center, 1515 Holcombe Blvd, Houston, TX, 77030, USA; 6 Department of Pulmonary Medicine, The University of Texas MD Anderson Cancer Center, 1515 Holcombe Blvd, Houston, TX, 77030, USA

**Keywords:** BRAF, dabrafenib, trametinib, vemurafenib, squamous, sarcomatoid, anaplastic thyroid cancer, dedifferentiated, undifferentiated, targeted therapy, chemotherapy, NTRK

## Abstract

Anaplastic thyroid cancer is a rare and aggressive thyroid cancer with an overall survival measured in months. Because of this poor prognosis and often advanced age at presentation, these patients have traditionally been treated palliatively and referred for hospice. However, recent progress using novel therapies has energized the field, and several promising clinical trials are now available for these patients. This review will highlight this progress and the potential treatments that could pave the way to improved outcomes and quality of life for patients with this disease.

## Introduction

Anaplastic thyroid carcinoma (ATC) is a rare form of thyroid cancer and one of the most aggressive cancers in humans. In part because of ATC’s rarity and short overall survival, very little progress has been made in the treatment of this disease until recently. An understanding of the molecular aberrations in this disease has advanced the field, leading to one of the most important treatment discoveries—BRAF targeting in *BRAF V600E*LN876569-mutant ATC. While this is an exciting discovery and represents substantial progress, only 25–45% of ATCs harbor a *BRAF* mutation ^1– 3^, and this treatment is not curative. However, a renewed interest in this disease will likely lead to a deeper comprehension of the mechanisms by which these tumors survive, that one day may result in better therapies and eventually a cure for this devastating disease.

## Background

The usual clinical presentation of ATC is of a patient with a rapidly expanding neck mass that may lead to compressive symptoms such as dysphagia and dyspnea. Patients may also have hoarseness due to vocal cord paralysis secondary to laryngeal nerve paralysis that can progress to stridor. However, some patients may have few symptoms until the disease is quite advanced and, therefore, most patients with ATC are not surgical candidates. Furthermore, approximately 46% of newly diagnosed patients present with widely metastatic disease at diagnosis ^4^. Patients may give a past history of long-standing goiter, thyroid nodules, or prior thyroid cancer. According to one series, in more than 80% of cases, there is a co-existing differentiated thyroid cancer (DTC) giving rise to a less differentiated or undifferentiated tumor ^5^.

The diagnosis of ATC can be made on fine needle aspiration, core biopsy, or surgical resection material. Fine needle aspiration is often the first attempt at biopsy, since this is the standard of care for thyroid nodules and suspected thyroid cancers. However, if the diagnosis of ATC is suspected, a core needle biopsy is preferable in order to perform genetic mutational analysis of the tumor. Many patients are not suspected to have ATC and are diagnosed only after undergoing thyroidectomy when the surgical pathology is reviewed.

The differential diagnosis of ATC includes lymphoma, poorly differentiated thyroid carcinoma (PDTC), poorly differentiated medullary thyroid carcinoma (MTC), squamous cell carcinoma from an adjacent site, and metastasis from other solid tumors. The diagnosis can be challenging because of the frequent loss of thyroid (TTF-1 and PAX8) and epithelial cell markers (cytokeratins). Morphologically, ATC is highly variable, from sarcomatoid, squamoid, and epithelioid to pleomorphic/giant cell cytologic features. ATC often grows as discohesive sheets of overtly malignant cells with high-grade features including marked necrosis and mitoses. An associated mixed inflammatory component is also present in the background.

According to the American Joint Committee on Cancer (AJCC) thyroid cancer staging system ^6^, all ATC patients are considered to have stage IV disease owing to the extremely high mortality rate. Stage IV disease is further broken down into stage IVA (intrathyroidal and surgically resectable without distant metastatic disease), stage IVB (extrathyroidal, with or without lymph node metastases but without distant metastatic disease), and stage IVC (distant metastatic disease at presentation).

Overall prognosis for ATC remains poor despite recent advances. The median overall survival remains around 3–5 months with a 1-year survival of approximately 20% ^7^. Patient factors associated with poorer prognosis include advanced age (>60–70 years), male gender, presence of leukocytosis (>10,000), and symptoms (such as rapidly growing tumor, pain in the neck, dyspnea, dysphagia, and hoarseness). Histologically, larger tumor size (>5 cm), extrathyroidal invasion, giant cell and pleomorphic pattern, and/or presence of distant metastases are tumor factors that can portend a poor survival and predict treatment failure. Tumors with co-existing well-differentiated papillary thyroid carcinoma appear to fare better ^2, 7– 9^. In terms of treatment factors, patients with tumors that are surgically resectable and who are able to undergo external beam radiation have improved survival ^4, 8– 16^. The dose of external beam radiation is also prognostic, with patients who are able to undergo higher doses (>60 Gy) surviving longer, even those who have not undergone complete resection ^12, 17^. Somatic tumor mutations may also affect outcomes. ATC tumors with *TERT* promotor mutations had a significantly shorter overall survival, particularly when these co-existed with either *BRAF* or *RAS* mutations ^1^. In another study, ATC patients with *TP53* mutations were shown to have a trend towards shorter time to failure after primary treatment ^2^.

Standard treatment for patients with stage IVA disease and some IVB patients is curative intent surgery, as this has been associated with longer survival ^10, 14, 18– 20^. Surgery should be performed by a surgeon with experience in advanced thyroid cancer. Post-operative radiation with radiosensitizing chemotherapy should be started within 4 weeks of surgery. Chemotherapy regimens are detailed in the American Thyroid Association guidelines for the management of patients with ATC ^21^. Unfortunately, surgery is not possible in the majority of patients, as most present with locally advanced and invasive disease. In general, if the patient has unresectable IVB disease, chemoradiation can be considered with the goal of local control. If the radiation field is too large or the chemoradiation treatment deemed too morbid for the patient, systemic therapy can be considered in these patients, particularly the novel targeted treatments discussed in the next section. Patients with IVC disease are the most challenging to manage, as balancing the need to control both local and distant metastatic disease can be difficult. Treatment guidelines recommend palliative radiation to control locoregional disease; however, our experience has been that palliative radiation provides little control. Thus, our practice is to treat these patients on clinical trials (preferable) or outside a trial with novel targeted therapies. A better understanding of the genetics of thyroid cancers has led to the use of novel therapies for ATC, which is the theme of this manuscript.

## Genetics of anaplastic thyroid cancer

**Figure 1.**
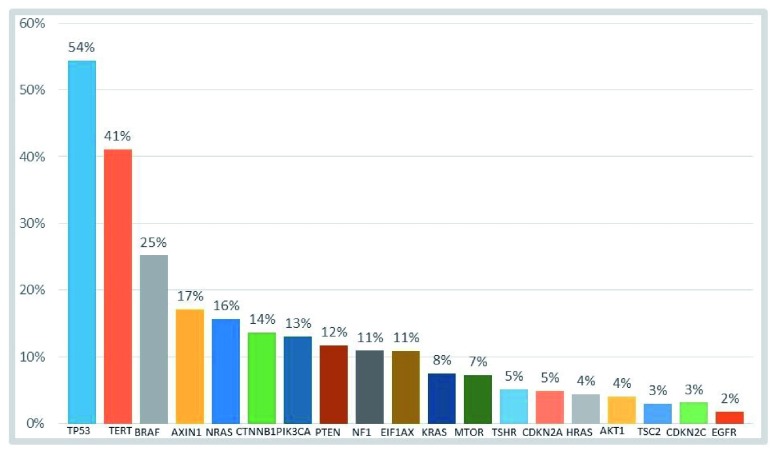
Distribution of oncogenic mutations in anaplastic thyroid carcinoma. Data compiled from three sources: COSMIC ( cancer.sanger.ac.uk) ^3^, Kunstman *et al.* ^24^, and Landa *et al.* ^1^. Genetic rearrangements are not represented on the graph.

The MAP kinase and PI3 kinase pathways are commonly activated in thyroid cancer. The common driver mutations that are seen in ATC are the same as those in DTC, as these tumors are usually derived from this class of tumor, particularly papillary, follicular, and Hurthle cell thyroid cancers. Thus, *BRAF*, *NRAS*, *KRAS*, and *HRAS* are common driver mutations seen in ATC. Mutations in tumor suppressor genes include *p53*, *NF1*, and *PTEN*. Secondary mutations such as *PIK3CA*, *TERT* promotor, *CTNNB1*, *EIF1AX*, *MTOR*, *CDKN2A*, and *AKT1* can co-exist with driver mutations and tumor suppressor gene mutations. Figure 1 shows the frequency of point mutations in ATC. The genetic rearrangements observed in papillary thyroid cancer, *RET*/ *PTC* and *NTRK*, have also been described in ATC. Most recently, *ALK* and *ROS1* genetic rearrangements have also been described ^22, 23^.

**Figure 2.**
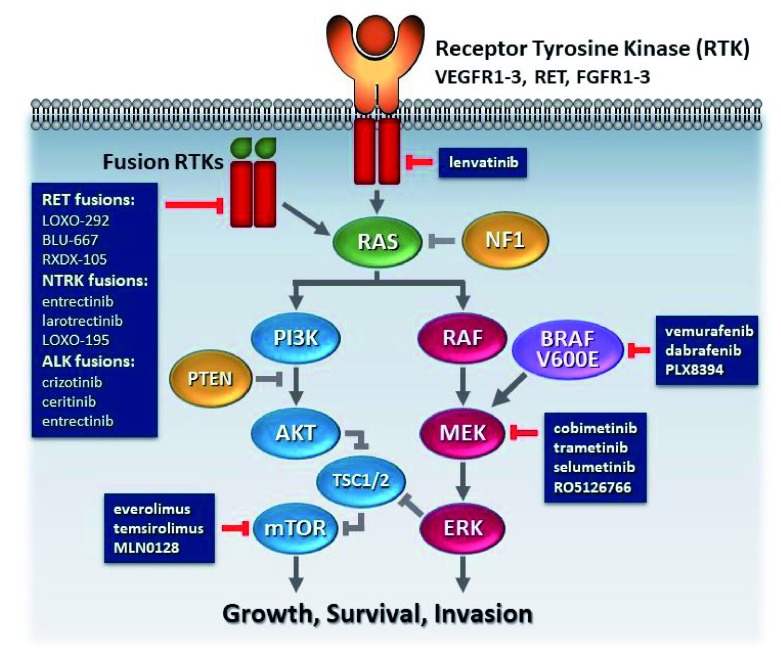
Schematic overview of the two most common pathways with genetic alterations in thyroid cancer—mitogen-activated protein kinase (MAPK) and phosphatidylinositide 3-kinase (PI3K) pathways. The pathways can be activated by receptor tyrosine kinases (RTKs) being activated by growth factors or by overexpression of the receptor. Constitutive activation of the signaling pathways can be caused by mutations along the pathway, such as BRAF, RAS, PI3K, AKT, or mammalian target of rapamycin (mTOR), mutations in tumor suppressor genes, such as neurofibromin 1 (NF1) or phosphatase and tensin homolog (PTEN) mutations, or fusion RTKs, such as those in RET, neurotropic tropomyosin receptor kinase (NTRK), or anaplastic lymphoma kinase (ALK). Drugs that can inhibit at critical points in the pathway are listed in grey boxes. FGFR, fibroblast growth factor receptor; TSC1, tuberous sclerosis complex 1; TSC2, tuberous sclerosis complex 2; VEGFR, vascular endothelial growth factor receptor.

Of all of the genetic mutations that occur in ATC, *BRAF V600E* is the most common (25–48% ^1– 3, 24^) actionable mutation, and therefore the most is known regarding novel therapies that target this mutation in ATC. However, other rare mutations and genetic aberrations may also prove to be actionable, such as *ALK* translocations. Unfortunately, at this time, the majority of mutations in ATC are not actionable, but targeting of the tumor microenvironment or common pathways is an alternative approach. Figure 2 shows drugs and drug targets of interest in thyroid cancer.

## Novel and promising therapies for ATC

### Targeting genetic aberrations

***BRAF inhibitors*.** Several case reports of responses in *BRAF*-mutated ATC to selective BRAF inhibitors sparked interest in using these drugs in this disease ^25– 27^. Two basket trials that included *BRAF*-mutant ATC have been reported. The first trial reported had a very small cohort of ATC patients (n=7) treated with vemurafenib ^28^. In this study, one complete response (CR) and one partial response (PR) were reported. These two patients had relatively long responses and survival, as one was alive at the time of data cut-off after 12.7 months and the other lived for 15.5 months after starting the trial. Vemurafenib is FDA approved for use in the treatment of melanoma.

A basket trial with the selective BRAF inhibitor dabrafenib combined with the MEK inhibitor trametinib has, by far, reported the largest cohort of BRAF inhibitor-treated ATC patients ^29^. This trial was designed to enroll *BRAF*-mutated patients with nine different histologies, including an ATC cohort. Cohorts showing promising results were expanded. A total of 16 *BRAF*-mutated ATC patients, all previously treated with radiation or surgery, were reported; 11 of the 16 patients achieved PR. The median PFS and OS were not reached but were estimated to be 79% and 80% at 1 year, respectively. The most common adverse events were fatigue, pyrexia, and nausea. The combination of dabrafenib and trametinib is approved for *BRAF*-mutated melanoma and squamous cell carcinoma of the lung and has received Breakthrough Therapy Designation by the Food and Drug Administration for ATC.

The advantage of selective BRAF/MEK inhibitors is how rapidly these drugs can start to take effect. We previously published a case report of a *BRAF*-mutated ATC patient who failed several lines of therapy ^26^. The patient had stridor from significant airway obstruction prior to starting compassionate-use dabrafenib plus trametinib. This treatment prevented tracheostomy, and imaging performed 1 month after initiating treatment showed a dramatic response to therapy with a patent airway. Another way that the rapid onset of this combination therapy can be harnessed is by using these drugs as neoadjuvant therapy to shrink the tumor prior to surgery. Neoadjuvant vemurafenib has been studied in papillary thyroid cancer, and this strategy was deemed safe ^30^. In ATC, neoadjuvant BRAF plus MEK inhibitors could prove to be useful in patients with stage IVB disease, leading to regression and thereby allowing for complete surgical resection. Further studies are needed to determine if neoadjuvant treatment will result in improved locoregional control and/or survival.

***NTRK inhibitors*.** There are two NTRK inhibitors at this time in clinical trials. Larotrectinib (LOXO) is a selective pan-TRK inhibitor that is highly potent against TRKA, B, and C. Trial results of three separate studies that included a total of 55 patients with *NTRK* fusions were reported at the American Society of Clinical Oncology (ASCO) Annual Meeting in 2017. There were 17 unique tumor types, including 9% thyroid cancer patients. The response rate in the 55-patient cohort was 78%, with four thyroid cancer patients achieving PR and one achieving CR. The histology of these five thyroid cancer patients was not reported. The drug was deemed tolerable, with fatigue, dizziness, anemia, and vomiting being the most common adverse events. Entrectinib (RXDX-101) is another pan-TRK inhibitor but also targets *ALK* and *ROS1* fusions. The drug is being studied in a basket trial for patients with *NTRK*, *ALK*, or *ROS* genetic rearrangements (NCT02568267). Results from the phase I trial have been reported; however, there were no thyroid cancer patients treated on this trial ^31^. Central nervous system activity was reported in one patient with *NTRK* fusion lung cancer who achieved CR in the brain.

***Alk and ROS1 inhibitors*.** *ALK* mutations and fusions are uncommon events in thyroid cancer. They have been reported in 1.6% of papillary thyroid cancers, 4% of ATCs, and 9% of PDTCs in one series ^22^. Only one case of a thyroid cancer patient with *ALK* fusion treated with an ALK inhibitor has been reported in the literature. The patient was treated with crizotinib after failing standard therapy for ATC and achieved a remarkable response ^32^. A trial with ceritinib for *ALK* mutation or fusion is underway (NCT02289144). Only one case of *ROS1* rearrangement in papillary thyroid cancer has been reported. Although it has not been reported in ATC, it is possible that these exist in patients with ATC transformed from papillary thyroid cancer. Entrectinib (discussed above) is a pan-TRK, ALK, and ROS1 inhibitor that will be investigated in thyroid cancer patients in a currently enrolling basket trial (NCT02568267).

***mTOR inhibitors*.** Targeting of *MTOR*, which is in the PI3 kinase pathway, has proven successful in other solid tumors such as renal cell carcinoma. Activating mutations along this pathway ( Figure 1), including *PIK3CA* and *MTOR*, as well as tumor suppressor loss of regulatory function mutations in *PTEN*, *TSC1*, and *TSC2*, are seen in ATC. *NF1* is another potential target of mTOR inhibitors, as *NF1*-mutated tumors can signal down the PI3 kinase pathway. Everolimus is the mTOR inhibitor most studied in thyroid cancer ^33– 35^. Several of these trials have included small numbers of ATC patients, but only occasional responses have been seen to date. One very impressive response was seen in a patient with a *TSC2* mutation. This patient responded to everolimus for 18 months before developing progression. Analysis of the tumor at progression revealed a new *MTOR* mutation ^36^. A study of a second-generation mTOR inhibitor with broad-spectrum inhibition of the mTOR complex is currently enrolling ATC patients (NCT02244463).

### Targeting the tumor microenvironment

***Antiangiogenics*.** Antiangiogenics are effective in DTCs and MTCs, and lenvatinib, sorafenib, vandetanib, and cabozantinib are FDA approved for these indications. These and other antiangiogenics ^37– 40^ have also been studied in ATC, but only one antiangiogenic drug, lenvatinib, has shown sufficient efficacy to warrant further study as a single agent in a phase II trial. Lenvatinib was studied in a trial that included all three thyroid cancer types in Japan ^41^. This trial enrolled 17 ATC patients. Four patients (24%) achieved PR, 12 (71%) had stable disease, and one (6%) had progression as their best response. The median PFS in the ATC cohort was 7.4 months (95% CI: 1.7–12.9) and the median OS was 10.6 months (95% CI: 3.8–19.8). However, only 10 of the 17 tumors were centrally confirmed to be ATC. The favorable results of this study led to a larger, international, phase II trial to test the efficacy of lenvatinib in ATC (NCT02657369).

Another interesting trial that holds promise is a study with intensity modulated radiotherapy with radiosensitizing chemotherapy plus or minus pazopanib (randomly assigned; NCT01236547). This study—the first trial in ATC to complete anticipated enrollment—closed to new patient enrollment in 2016. The primary endpoint is OS; thus, the results are not expected until 2018.

***Immunotherapy*.** At this time, there are no trial reports using immunotherapy in ATC. However, many immunotherapy trials for ATC are currently recruiting patients. Immunoprofiling of ATC tumors has shown that tumor-infiltrating lymphocytes are present in high numbers and that these tumors express programmed death-ligand 1 (PD-L1), which plays a role in suppressing the immune system ^42– 46^. Previous small studies have shown that tumor-associated macrophages (TAMs) are present in high frequencies in ATC compared with PDTC and DTC. Increased TAMs are associated with more invasive cancers and decreased survival, suggesting a tumorigenic role of TAMs in these cancers ^47– 49^. Although limited data exist with regard to functional characterization of TAMs, it is thought that TAMs closely resemble M2 (protumoral macrophages). In other cancer types, it has been shown that TAMs express programmed cell death-1 (PD-1), suggesting that PD-1/PD-L1 therapies may also function through a direct effect on macrophages ^50^. Additionally, TAM-focused therapeutic strategies could synergize with immunotherapy.

Several checkpoint inhibitors that target PD-1 or its ligand, PD-L1, are approved for other cancers. However, given our experience, because of the rate at which ATC tumors expand and the usually large size of ATC tumors, we believe that these drugs will require combination therapy with kinase inhibitors, cytotoxic chemotherapy, or radiation. In an immunocompetent mouse model of *BRAF*/ *p53*-mutated ATC, the combination of a BRAF inhibitor plus anti-PD-L1 drugs led to far better responses than either drug alone ^51^, suggesting that combination therapy should be pursued in clinical trials. A clinical trial using this approach is currently underway (NCT03181100), as are other trials using combination checkpoint inhibitors (NCT03246958) and radiation plus immunotherapy (NCT03122496 and NCT03211117).

## Summary of the evolving management and future directions in ATC

**Figure 3.**
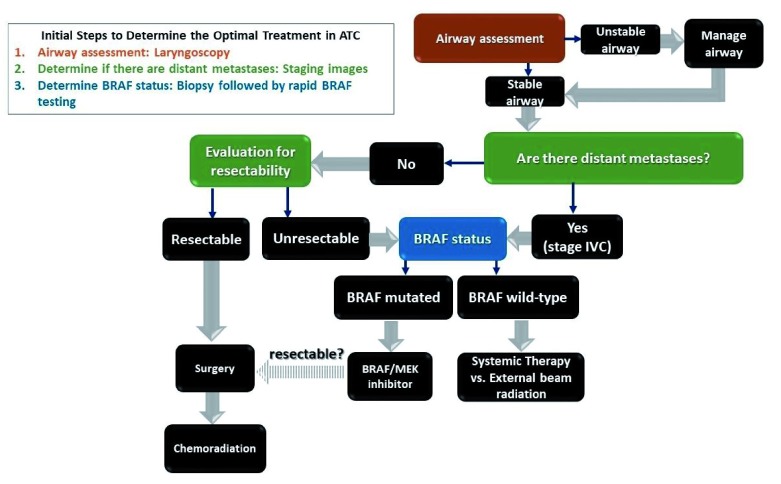
Proposed algorithm to determine best treatment in anaplastic thyroid carcinoma (ATC). The most critical steps that lead to important decision points are in colored boxes and should be performed in tandem. Brown box: the airway should also be assessed by laryngoscopy. An unstable airway will require immediate management, which might include steroids, intubation, tracheostomy, or hospice if the patient has a poor performance status. Green boxes: full staging with cross-sectional, contrast-enhanced imaging of the brain, neck, chest, and abdomen as well as bone scan or PET/CT (preferable) should be performed in order to determine if there are distant metastases. If brain metastases are present, these may require radiation or surgery prior to any therapy. Based on the staging images and the patient’s performance status, the patient should be evaluated to determine if the tumor is resectable and a good surgical candidate. Blue box: a rapid test to determine *BRAF* status should be performed as soon as possible. Patients with a stable airway can be triaged to BRAF-directed therapy if a *BRAF V600E* mutation is present. If there is a treatment response that leads to resectability, surgery by an experienced surgeon may be considered for patients with low burden or no distant metastatic disease. In the absence of a *BRAF V600E* mutation, external beam radiation to the neck or systemic therapy on clinical trial is recommended. The decision between systemic therapy and external beam radiation in the *BRAF* wild-type subgroup will depend on local versus distant disease burden.

Owing to the staggeringly high failure rate in ATC and recent renewed interest in this disease, new paradigm shifts are rapidly evolving and being adopted by many leaders in the field. While routine molecular testing of ATC tumors is not currently in treatment guidelines and is controversial, our group feels strongly that all of these patients’ tumors should be tested upon diagnosis, at minimum, to determine BRAF status. Patients with *BRAF* mutations can be offered treatment with combination selective BRAF plus MEK inhibitor therapy. Some patients with initially unresectable disease may become resectable and could be considered for surgery if they respond to BRAF/MEK combination therapy. Patients who are well enough and want to consider clinical trials should have a broader panel of mutations and fusions interrogated in order to identify those who would be appropriate for selective inhibitor trials such as the selective NTRK or RET inhibitor trials. Other driver mutations that are identified may help identify a clinical trial that is appropriate for the patient, particularly as more targeted therapies become available. Figure 3 shows a proposed algorithm for selecting treatment in ATC patients and how the molecular information of the tumor can be used to help guide therapy, particularly in patients with a *BRAF V600E* mutation, since currently the most promising treatments are in these patients.

However, in order for patients to begin therapy before they deteriorate clinically, it is necessary to be able to rapidly test patients for pertinent mutations. The gold standard of molecular testing with next-generation sequencing can take over 1 month before results are known (far too long for patients with a rapidly lethal disease). Thus, the use of liquid biopsy, the testing of blood for circulating cell-free tumor DNA, which has been shown to be highly sensitive for identifying *BRAF* mutations in circulating DNA, should be employed routinely in patients who do not have available tissue specimens ^52^. In those with available tissue, immunohistochemistry for BRAF V600E on surgical specimens or core needle biopsy has proven to be a useful, rapid tool to identify BRAF-mutated ATC patients ^53^.

Resistance to kinase inhibitors remains a major obstacle in the treatment of ATC. Thus, moving away from single-agent therapy for ATC is one step in the right direction. Combinations of targeted therapy with immunotherapy may help overcome resistance and are currently being studied in clinical trials. Radiation plus systemic therapy with either immunotherapy or targeted therapies are also promising strategies for ATC patients. Also, due to the very high recurrence rate after definitive treatment with surgery and radiation, a systemic adjuvant approach should be studied in this setting.

The future is much more promising for ATC patients; however, many physicians continue to recommend hospice as the only option for these patients. Our hope in the current environment with potential promising targeted therapies is that more patients are aware of and will participate in clinical trials, particularly those whose tumors are molecularly driven. Increasing medical and public awareness of expanding clinical trial options for ATC, coupled with enhanced availability of tumor sequencing, may prime a cycle for rapid enrollment and outcome data in this rare disease.
